# Purification of cardiomyocytes and neurons derived from human pluripotent stem cells by inhibition of *de novo* fatty acid synthesis

**DOI:** 10.1016/j.xpro.2022.101360

**Published:** 2022-04-28

**Authors:** Sho Tanosaki, Tomohiko Akiyama, Sayaka Kanaami, Jun Fujita, Minoru S.H. Ko, Keiichi Fukuda, Shugo Tohyama

**Affiliations:** 1 Department of Cardiology, Keio University School of Medicine, Shinjuku, Tokyo 160-8582, Japan; 2 Department of Systems Medicine, Keio University School of Medicine, Shinjuku, Tokyo 160-8582, Japan; 3 Heartseed Inc., Shinjuku, Tokyo 160-0015, Japan

**Keywords:** Cell culture, Cell isolation, Stem Cells, Cell Differentiation

## Abstract

Here we describe a protocol to obtain highly pure cardiomyocytes and neurons from human induced pluripotent stem cells (hiPSCs) via metabolic selection processes. Compared to conventional purification protocols, this approach is easier to perform and scale up and more cost-efficient. The protocol can be applied to hiPSCs and human embryonic stem cells.

For complete details on the use and execution of this protocol, please refer to Tohyama et al. (2016) and Tanosaki et al. (2020).

## Highlights

• Metabolic selection of hPSC-CMs with lactate and orlistat treatment
• Metabolic selection of hPSC-derived neurons with orlistat treatment
• Protocol can be used with hiPSCs or hESCs

## Graphical abstract

**Figure:**
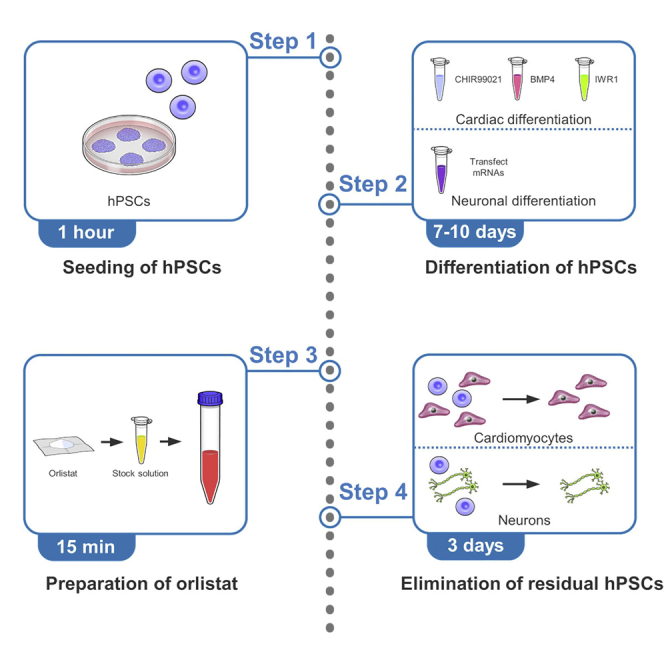

## Before you begin

The first part of this manuscript describes the metabolic selection processes for cardiomyocytes (CMs), and the latter part describes the process for neurons. The protocol described below has been used in our laboratory for years with multiple cell lines. Human induced pluripotent stem cells (hiPSCs) and human embryonic stem cells (hESCs) upregulate *de novo* fatty acid synthesis pathways, enabling rapid proliferation. Inhibition of fatty acid synthase (FASN) by its inhibitor orlistat induces cell death in residual human pluripotent stem cells (hPSCs). Following orlistat treatment, hPSC-derived CMs (hPSC-CMs) are cultured in glucose and glutamine-depleted with lactate medium for additional purification to eliminate differentiated non-CMs (Tohyama et al., 2013, Tohyama et al., 2016, Tohyama et al., 2017). Metabolic selection using lactate enhances the maturation of hPSC-CMs (Nakano et al., 2017, Ni et al., 2021, Tohyama and Fukuda, 2017).

hiPSCs and hESCs can be obtained from academic organizations, such as the Center for iPS Cell Research and Application (CiRA), Kyoto University, or commercially purchased. All experiments using hPSCs must follow institutional guidelines and applicable domestic laws and regulations. All procedures were performed under sterile conditions unless otherwise specified.

It is important to keep the passage number of the cells as low as possible, as excessive passaging may cause abnormal karyotypes and alter the phenotype, which can influence differentiation.

### Preparation of Matrigel

**Timing: 2 days**

1. Thaw Matrigel® Growth Factor Reduced (GFR) Basement Membrane Matrix at 4°C overnight.
2. Cool 1.5 mL microcentrifuge tubes on ice or in an ice-cold thermal block.
3. Transfer the bottle of Matrigel from the refrigerator to ice.
4. Aliquot the Matrigel.
  a. We usually prepare 682 μL aliquots (∼10 mg/mL).
5. Store the aliquots at −30°C. Based on our experience, Matrigel aliquots may be stored for up to 6 months.

**CRITICAL:** Matrigel quickly becomes gel-like. To avoid this, the bottle of Matrigel must be transferred from the refrigerator on ice.

## Key resources table

**Table:** 

REAGENT or RESOURCE	SOURCE	IDENTIFIER
**Antibodies**		

OCT4 mouse monoclonal antibody	Santa Cruz Biotechnology	sc-5279
OCT4 rabbit monoclonal antibody	Abcam	Ab200834
Anti-Cardiac Troponin T-FITC	Miltenyi Biotec	130-119-575
REA Control(I)-FITC	Miltenyi Biotec	130-118-354
α-actinin	Sigma-Aldrich	A7811
βIII tubulin	Cell Signaling Technology	5666
Donkey anti-mouse IgG (H+L) highly cross-adsorbed secondary antibody, Alexa Fluor 594	Thermo Fisher Scientific	A21203
Donkey anti-mouse IgG (H+L) highly cross-adsorbed secondary, Alexa Fluor 488	Thermo Fisher Scientific	A21202
Goat anti-mouse IgG (H+L) highly cross-adsorbed secondary, Alexa Fluor 546	Thermo Fisher Scientific	A11030
Goat anti-rabbit IgG (H+L) cross-adsorbed secondary, Alexa Fluor 488	Thermo Fisher Scientific	A11008
Donkey anti-rabbit IgG (H+L) highly cross-adsorbed secondary, Alexa Fluor 546	Thermo Fisher Scientific	A10040

**Chemicals, peptides, and recombinant proteins**		

DMEM/F12 GlutaMAX	Thermo Fisher Scientific	10565-018
StemFit AS103C	Ajinomoto	N/A
StemFit AK02N	Ajinomoto	AK02N
StemFit AS501	Ajinomoto	N/A
RPMI 1640	FUJIFILM Wako Pure Chemical	189-02025
MEMα	Thermo Fisher Scientific	12571-048
Opti-MEM™ I Reduced Serum Medium	Thermo Fisher Scientific	31985070
Bovine Albumin Fraction V (7.5% solution)	Thermo Fisher Scientific	15260037
D-PBS	FUJIFILM Wako Pure Chemical	045-29795)
Y-27632	FUJIFILM Wako Pure Chemical	034-24024
TrypLE Select	Thermo Fisher Scientific	12563-011
Matrigel® Growth Factor Reduced (GFR) Basement Membrane Matrix	Corning	354230
iMatrix-221	Matrixome	892 061
iMatrix-511	Matrixome	892 011
B-27™ supplement, minus insulin	Thermo Fisher Scientific	A1895601
Fetal bovine serum chile, USDA approved	Biowest	S1560-500
2.5 g/L Trypsin EDTA	NACALAI TESQUE	35554-64
Lipofectamine™ Messenger MAX™	Thermo Fisher Scientific	LMRNA001
Dimethyl sulfoxide (DMSO)	Sigma-Aldrich	D2650-100ML
Orlistat	Sigma-Aldrich	O4139-25MG
Hoechst 33342	Thermo Fisher Scientific	H3570
DAPI	Dojindo	D523

**Experimental models: Cell lines**		

253G4	Center for iPS Cell Research and Application (CiRA), Kyoto University	N/A
201B7	CiRA, Kyoto University	N/A
H9	WiCELL	WA09
FfI14	CiRA, Kyoto University	https://www.cira-foundation.or.jp/e/project/homozygous.html

**Oligonucleotides**		

NEUROG2	(Zhou et al., 2014)	N/A

**Recombinant DNA**		

NEUROG2 and 3′UTR DNA	(Goparaju et al., 2017)	N/A

**Software and algorithms**		

ImageJ	National Institutes of Health	https://imagej.nih.gov/ij/
cellSens	Olympus	https://www.olympus-lifescience.com/en/software/cellsens/

**Other**		

4% Paraformaldehyde	Muto Pure Chemicals	33111
Triton X-100	Sigma-Aldrich	T9284
ImmunoBlock	KAC	CTKN001
LIVE/DEAD® Viability/Cytotoxicity Kit for Mammalian Cells	Thermo Fisher Scientific	L3224
Leukocyte Alkaline Phosphatase Kit	Sigma-Aldrich	86R-1KT

## Materials and equipment

**Table:** 

Reagent	Final concentration	Amount
Bovine Albumin (BSA) Fraction V (7.5% solution)	0.1%	100 μL
D-PBS	N/A	7.4 mL
Total	N/A	7.5 mL

Use immediately after preparation.

**Table:** 

Reagent	Final concentration	Amount
Y-27632	10 mM	25 mg
0.1% BSA	N/A	7.39 mL
Total	N/A	7.39 mL

Store at −30°C for up to 6 months.

**Table:** 

Reagent	Final concentration	Amount
RPMI-1640	N/A	500 mL
B27 minus insulin	N/A	10 mL
Total	N/A	510 mL

Store at 4°C for up to 1 month.

**Table:** 

Reagent	Final concentration	Amount
CHIR-99021	12 mM	5 mg
DMSO	N/A	900 μL
Total	N/A	900 μL

Store at −30°C for up to 6 months.

**Table:** 

Reagent	Final concentration	Amount
IWR-1	10 mM	25 mg
DMSO	N/A	6.1 mL
Total	N/A	6.1 mL

Store at −30°C for up to 6 months.

**Table:** 

Reagent	Final concentration	Amount
BMP4	10 μg/mL	50 μg
0.1% BSA	N/A	5 mL
Total	N/A	5 mL

Store at −30°C for up to 6 months.

**Table:** 

Reagent	Final concentration	Amount
Orlistat	12 mM	25 mg
DMSO	N/A	4.2 mL
Total	N/A	4.2 mL

Store at −30°C for up to 6 months.

**Table:** 

Reagent or resource	Source	Identifier
Falcon® 100 mm TC-treated Cell Culture Dish	Falcon	353003
Falcon® 6-well Clear Flat Bottom TC-treated Multiwell Cell Culture Plate, with Lid	Falcon	353046
Falcon® 12-well Clear Flat Bottom TC-treated Multiwell Cell Culture Plate, with Lid	Falcon	353043
Falcon® 24-well Clear Flat Bottom TC-treated Multiwell Cell Culture Plate, with Lid	Falcon	353047

**CRITICAL:** Solutions must be prepared in a sterile environment.

***Alternatives:*** AS103C and AK02N are culture media designed to maintain undifferentiated hPSCs. Both lack lipids other than essential lipids; however, AS103C contains a higher concentration of tryptophan to stimulate cell proliferation (Someya et al., 2021). AS103C can be substituted with AK02N (Ajinomoto) or mTeSR1 (STEMCELL Technologies). AS501 is a culture medium designed for metabolic purification using lactate and thus can be substituted with DMEM without glucose, glutamine, or phenol red (A1443001, Thermo Fisher Scientific) supplemented with L-lactic acid (129-02666, FUJIFILM Wako Pure Chemical) (final concentration: 4 mM) and bovine albumin fraction V (15260037, Thermo Fisher Scientific) (final concentration: 0.1%).

## Step-by-step method details

### Thawing hPSCs

**Timing: 2 h**

This step describes methods to coat culture apparatus with Matrigel and to thaw frozen hPSCs.

1. Place the culture apparatus of your choice on the bench.
  a. We usually use a 100 mm culture dish for the maintenance of hPSCs and differentiation to CM.
2. Thaw Matrigel cooled to 4°C into precooled DMEM/F12 medium.
  a. We usually thaw 682 μL of Matrigel into 42 mL of DMEM/F12 medium for seven 100 mm culture dishes.
  b. Matrigel may be thawed from frozen state by applying small amount (i.e., 1 mL) of DMEM/F12 medium to aliquots. Make sure that Matrigel is dissolved completely without making a lump.
3. Apply Matrigel diluted in DMEM/F12 medium to the culture apparatus.
  a. We usually apply 6 mL of Matrigel diluted in DMEM/F12 medium per 100 mm culture dish (culture area: 55 cm^2^). However, the amount of Matrigel diluted in DMEM/F12 medium can be adjusted for each culture apparatus according to the culture area.
  b. Tilt the culture apparatus to ensure that Matrigel uniformly covers the culture area.
4. Incubate the culture apparatus to which Matrigel was applied at room temperature for 1 h.
5. During the previous step, add Y-27632 to 30 mL StemFit AS103C medium for one 100 mm dish. (Final concentration: 10 μM).
6. Warm the vial containing hPSCs in a 37°C water bath. In most cases, when the frost on the vial is just before completely dissolved is the optimal time to end warming.
7. Disinfect the vial with 70% EtOH and collect the cells with 10 mL of StemFit AS103C supplemented with 10 μM Y-27632.
8. Centrifuge the cells at 300 × *g* for 4 min.
9. Aspirate the supernatant and resuspend the cells in 10 mL of StemFit AS103C supplemented with 10 μM Y-27632.
  ***Optional:*** Determine cell number and viability using any laboratory method. We used a Vi-CELL XR cell counter (Beckman Coulter Inc.).
  a. 2.0 × 10^6^ cells are sufficient for a 100 mm dish.
10. Aspirate the Matrigel diluted in DMEM/F12 medium and apply cells resuspended in StemFit AS103C supplemented with 10 μM Y-27632.
11. Gently rock the culture apparatus to allow the cells to disperse uniformly.
12. Gently place the culture apparatus in an incubator at 37°C. Gently rock the culture apparatus again to ensure an even distribution of cells.

**CRITICAL:** All procedures must be performed using standard precautions on a clean bench to avoid contamination unless specified.

**CRITICAL:** Matrigel quickly becomes gel-like. To avoid this, Matrigel must be transferred from the refrigerator to an ice-cold thermal block. After thawing, the thawed Matrigel with DMEM/F12 medium should be cooled on ice or stored at 4°C. Cells usually attach to the culture apparatus within 1 h, but they can be easily detached by an external force. Do not move or touch the culture apparatus for at least 24 h.

### Maintenance of hPSCs

**Timing: 2–8 weeks**

13. Replace the culture medium with StemFit AS103C without Y-27632 every 24–48 h.
  a. Changing the culture medium every 48 h is sufficient, but when the cells reach 80% confluence, the culture medium should be changed every 24 h.
14. The cells should be observed every day. When the cells in the culture apparatus reach 80% confluence, the cells should be passaged.
15. Coat the culture apparatus of your choice as described in steps 1–4.
16. Warm StemFit AS103C in a water bath at 37°C.
17. Add Y-27632 to AS103C medium. (Final concentration: 10 μM).
18. Aspirate the culture medium and rinse the cells with 10 mL of D-PBS.
  a. The amount of D-PBS required may vary depending on the surface area of the culture apparatus. For one 100 mm dish, 10 mL of D-PBS is adequate.
19. Apply 500 μL of TrypLE Select. The amount of TrypLE Select may vary depending on the surface area of the culture apparatus.
20. Incubate the cells at 37°C for 3 min.
21. Gently tap the culture apparatus to facilitate cell detachment.
22. Collect the cells with 9.5 mL of AS103C supplemented with 10 μM Y-27632.
23. Centrifuge the cells at 300 × *g* for 4 min.
24. Aspirate the supernatant and resuspend the cells in StemFit AS103C supplemented with 10 μM Y-27632.
  a. Determine the cell number and viability using any laboratory method. We used a Vi-Cell XR Cell Counter (Beckman Coulter Inc.).
25. Aspirate the Matrigel diluted in DMEM/F12 medium and apply cells resuspended in StemFit AS103C with 10 μM Y-27632.
  a. For the maintenance of human pluripotent stem cells, we usually apply 0.5 × 10^5^ cells per 100 mm culture dish. However, the optimal number of cells needed may vary depending on the cell clone, passage, and culture apparatus.
  b. For differentiation into CMs, we usually apply 8.25 × 10^5^–11 × 10^5^ cells per 100 mm culture dish.
26. Gently rock the culture apparatus to allow the cells to disperse uniformly.
27. Gently place the culture apparatus in an incubator at 37°C. Gently rock the culture apparatus again to ensure an even distribution of cells. Go back to step 13 for maintenance of the cells.

**CRITICAL:** If cells become overly confluent, their pluripotent status and viability may be affected. Observe the cells periodically to ensure that they are undifferentiated and not overly confluent. If any differentiated cells or masses are observed, it is best to discard them and start all over again.

### Differentiation to hPSC-CMs

**Timing: 2 weeks**

This section describes the production of hPSC-CMs based on previous reports (Lian et al., 2013; Tohyama et al., 2016, 2017).

28. When the cells reach > 90% confluence, they are ready for cardiac differentiation. We usually induce differentiation to hPSC-CMs in 100 mm dishes, but 6-well plates work just as efficiently.
  a. Cells must be passaged at least once after thawing.
  b. The optimal start day of cardiac differentiation is day 4 after passage. Y-27632 must be removed at least 24 h before the initiation of cardiac differentiation.
29. For cardiac differentiation, the culture medium should be warmed to 37°C in a water bath.
30. Day 0: Dilute the stock solution of CHIR-99021 and BMP4 in RPMI-1640 medium with B27 minus insulin.
  a. We usually use 6 μM CHIR-99021 and 1 μg/mL BMP4.
31. Rinse cells with D-PBS, and change the medium to RPMI-1640 supplemented with B27 minus insulin, CHIR-99021, and BMP4.
  a. D-PBS must be removed immediately after its application to the cell.
32. On day 1 (24 h), rinse cells with D-PBS and change the medium to RPMI-1640 supplemented with B27 minus insulin.
  a. D-PBS must be removed immediately after its application to the cell.
33. On day 3, add 5 μM IWR-1 to RPMI-1640 medium supplemented with B27 minus insulin. Rinse the cells with D-PBS and change the medium to RPMI-1640 supplemented with B27 minus insulin and 5 μM IWR-1.
  a. D-PBS must be removed immediately after its application to the cell.
34. On day 6, rinse cells with D-PBS and change the medium to RPMI-1640 with B27 minus insulin.
  a. D-PBS must be removed immediately after its application to the cell.
35. 24 h later (day 7), change the medium to MEMα supplemented with 5% FBS.
  a. Rinsing with D-PBS is not necessary for this step.
36. On day 10, replace the culture medium with MEMα supplemented with 5% FBS.
  a. Rinsing with D-PBS is not necessary for this step.

***Note:*** Make sure to rinse the cells with D-PBS in the steps mentioned. Based on our experience, washing the cells with D-PBS improves differentiation efficiency. The critical factors affecting the success of cardiac differentiation are the status of hPSCs (pluripotency), cell number, and concentration of CHIR-99021. The optimal cell number and concentration of CHIR-99021 must be determined in a pilot experiment. In addition, the most critical phase of this differentiation protocol is the initial phase. It is important to treat cells with CHIR-99021 for at least 24 h. CHIR-99021 treatment induces a non-negligible amount of cell death, but it is a commonly observed phenomenon. Self-beating hPSC-CMs may be seen as early as day 7; however, this is accompanied by low differentiation efficiency. When high differentiation efficiency is achieved (i.e., 90% efficiency), self-beating may not be observed until day 10.

### Metabolic selection of hPSC-CMs

**Timing: Several days**

Here, we describe the purification method for hPSC-CMs. Use CMs on day 10–12. Purification with reseeding of hPSC-CMs is recommended for better purification efficiency compared to purification without reseeding. Based on our experience, metabolic selection without reseeding results in significantly decreased yield caused by detachment of the cells.

**Figure 1:**
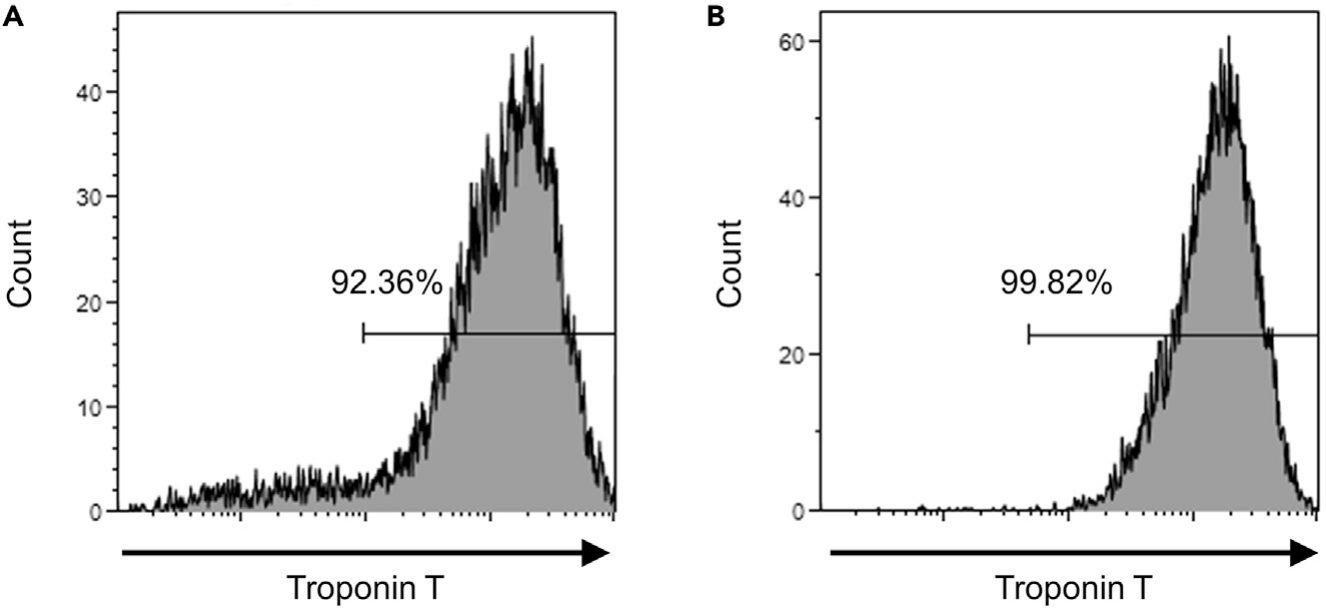
Representative flow cytometry histograms of cardiac Troponin T. (A and B) Representative images of flow cytometry before (A) and after (B) metabolic selection of hiPSC (253G4)-CMs. The cells are labeled with cardiac Troponin T.

***Note:*** The time period required for purification of hPSC-CMs (especially StemFit AS501) may vary according to the differentiation efficiency and the number of cells. In addition, if the cells are not completely dissociated into single cells when seeded, the purification may be disturbed. If non-CMs are observed, extend the treatment with StemFit AS501 until all non-CMs are successfully removed. This may take up to 7 days or more depending on the differentiation efficiency and cell number.

37. Warm D-PBS in a 37°C water bath.
38. Warm 2.5 g/L trypsin-EDTA in a 37°C water bath.
  a. Do not warm for an excessive time period, as trypsin may be inactivated by autodigestion. Always warm small amounts of trypsin.
39. Warm MEMα supplemented with 5% FBS in a 37°C water bath.
40. Add 6 μM orlistat to MEMα supplemented with 5% FBS.
41. Aspirate and replace the culture medium with pre-warmed D-PBS. Incubate the cells for 3 min in an incubator.
42. Remove D-PBS and add prewarmed 2.5 g/L trypsin-EDTA. Incubate cells for 5 min in an incubator.
  a. We usually use 1 mL of 2.5 g/L trypsin-EDTA for a 100 mm culture dish. Tilt and tap the dish gently to ensure uniform distribution of the trypsin-EDTA solution.
  b. Gently tap the culture apparatus to enhance cell detachment at 2 min 30 s.
  c. The detachment of the cells should be carefully observed to minimize trypsin treatment time. The treatment time may vary depending on the cell number and differentiation efficiency.
43. Collect the cells using MEMα supplemented with 5% FBS in a 15 mL conical tube.
  a. Before applying MEMα, pipette the cells with a p1000 micropipette to enhance dissociation. Avoid excess pipetting to minimize cell damage.
44. Centrifuge at 300 × *g* for 3 min.
45. Remove the supernatant and resuspend the cells in MEMα supplemented with 5% FBS and orlistat.
46. Apply the cell suspension to a collagen I-coated 150 mm culture dish.
  a. The optimal cell number for seeding may vary depending on the differentiation efficiency. We usually apply cells derived from two 100-mm culture dishes to one 150-mm culture dish.
47. Gently rock the culture apparatus to allow the uniform dispersion of cells.
48. Gently place the culture apparatus in an incubator at 37°C. Gently rock the culture apparatus again to ensure even distribution of the cells.
49. 72 h later, remove the culture medium and rinse the cells with D-PBS. Change the medium to StemFit AS501.
  a. D-PBS must be removed immediately after its application to the cell.
50. 48 h later, replace the medium with StemFit AS501.
  a. Carefully observe the cells to confirm successful elimination of non-CMs. This can be done by observing the non-beating cells by a phase-contrast microscope.
51. When the hPSC-CMs have been successfully purified, replace the medium with MEMα supplemented with 5% FBS.
52. Determine the purification efficiency using either immunocytochemistry or flow cytometry.
53. Immunocytochemistry.
  a. Rinse the cells with D-PBS and fix with ice-cold 4% paraformaldehyde (PFA) for 15–20 min at room temperature.
  b. Rinse the cells with D-PBS and treat with 0.1% Triton X-100 diluted in D-PBS for 5–15 min.
  c. Treat the cells with ImmunoBlock reagent for 30 min.
  d. Dilute primary antibodies against α-actinin (Sigma-Aldrich, A7811, 1:200) with ImmunoBlock.
  e. Apply the diluted antibodies to the cells and incubate overnight at 4°C.
  f. Remove the diluted antibodies and rinse the cells twice with D-PBS.
  g. Dilute the secondary antibody to 1:200. Donkey anti-mouse IgG (H+L) highly cross-adsorbed secondary antibody, Alexa Fluor 488 (Thermo Fisher Scientific, A21202) with ImmunoBlock.
  h. Apply the diluted antibody to the cells and incubate for 1 h at room temperature.
  i. Remove the secondary antibody and rinse the cells with D-PBS.
  j. Dilute the Hoechst 33342 stain (Thermo Fisher Scientific, H3570) to 1:2,000 in D-PBS.
  k. Apply the diluted Hoechst 33342 stain to the cells and incubate at room temperature for 1 h.
  l. Remove the Hoechst 33342 stain and apply D-PBS to the cells.
  m. Observe the cells under a microscope. If hPSC-CMs are successfully selected, only α-actinin-positive hPSC-CMs could be observed.
54. Flow cytometry (Figure 1).
  a. Replace the culture medium with 2 mL D-PBS for each well of a 6-well plate.
  b. Incubate the cells for 3 min at 37°C.
  c. Remove D-PBS and add 500 μL trypsin-EDTA.
  d. Incubate the cells for 3 min at 37°C. If the cells are not sufficiently detached, tap the culture apparatus and incubate the cells for a maximum of 2 more minutes.
  e. Collect the cells with MEMα medium supplemented with 5% FBS. Pipette the cells to ensure their complete dissociation (total 10 mL).
  f. Centrifuge at 300 × *g* for 3 min.
  g. Aspirate the supernatant and add 1 mL 4% PFA. Pipette the cells.
  h. Vortex for 20 min at room temperature.
  i. Add 10 mL D-PBS.
  j. Centrifuge at 300 × *g* for 3 min.
  k. Aspirate the supernatant and add 1 mL 0.1% Triton X-100 diluted in D-PBS.
  l. Vortex for 5 min at room temperature.
  m. Add 10 mL D-PBS.
  n. Centrifuge at 300 × *g* for 3 min.
  o. Aspirate the supernatant and add 1 mL ImmunoBlock reagent. Pipette the cells.
  p. Incubate for at least 30 min at 4°C.
  q. Divide 500 μL the suspended cells with ImmunoBlock reagent into two 1.5 mL microcentrifuge tubes and centrifuge at 300 × *g* for 5 min.
  r. Dilute the antibodies against cardiac Troponin T-FITC (Miltenyi, 130-119-575, 1:50) and REA Control(I)-FITC (Miltenyi, 130-118-354, 1:50) with ImmunoBlock reagent.
  s. Add the antibody and mix by gentle pipetting.
  t. Incubate 15 min at room temperature in the dark.
  u. Add 1 mL ImmunoBlock reagent.
  v. Centrifuge at 300 × *g* for 5 min.
  w. Aspirate the supernatant and add 350–400 μL D-PBS. Analyze the cells using a flow cytometer.

### Evaluation of the optimal dose and duration of orlistat treatment

**Timing: 2 weeks**

Here, we describe how to determine the optimal dose and duration of orlistat treatment, which are sufficient for eliminating undifferentiated hPSCs but do not significantly affect the viability of hPSC-CMs. This may vary depending on the cell line and growth speed.

**Figure 2:**
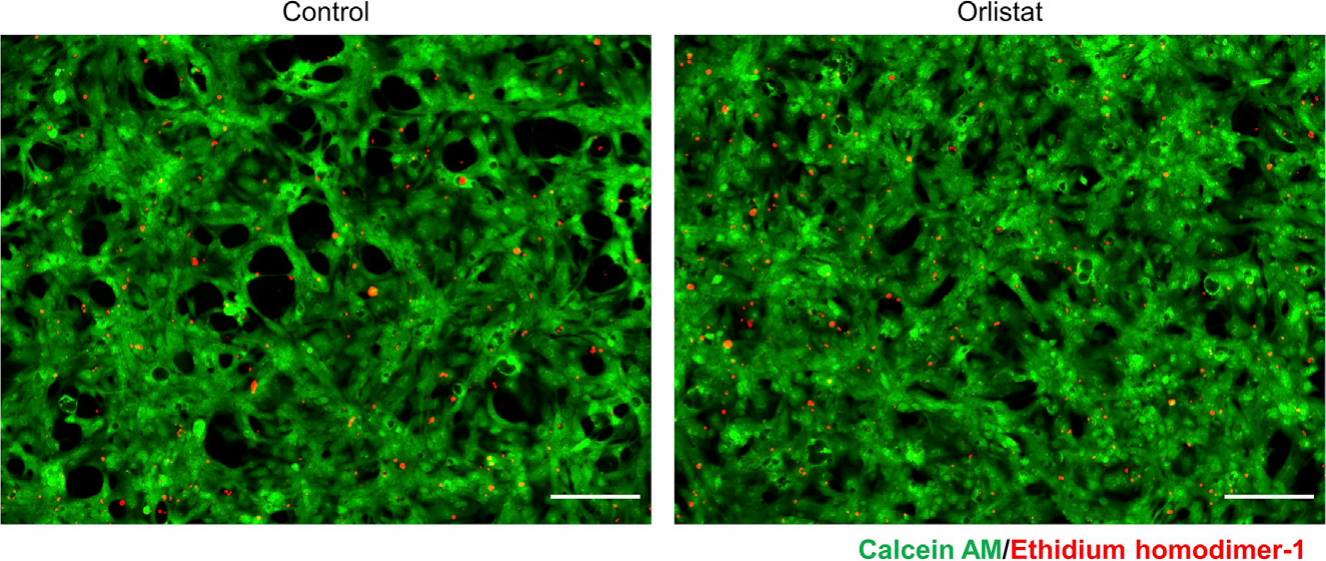
LIVE/DEAD Assay of hiPSC (253G4)-derived cardiomyocytes (hiPSC-CMs) treated with or without 6 μM orlistat for 72 h Orlistat treatment does not affect the viability of hiPSC- CMs. Scale bars: 200 μm.

55. On day 0, seed the hPSCs.
  a. The number of seeded cells should be adjusted so that the control (0 μM orlistat) reaches 90% confluence after 72 h of orlistat treatment.
56. On day 2, replace the culture medium with StemFit AS103C without Y-27632.
57. On day 4, dilute the orlistat stock solution with StemFit AS103C medium and prepare StemFit AS103C supplemented with 12 μM orlistat. For the control, the same amount of vehicle (DMSO) should be diluted with StemFit AS103C medium.
58. Prepare StemFit AS103C supplemented with 0, 2, 4, 6, 8, and 10 μM orlistat by mixing the medium prepared in the previous step.
59. Replace the culture medium with StemFit AS103C medium with or without orlistat prepared in the previous step.
60. Incubate the hPSCs for 24–72 h.
61. On day 5–7, observe the hPSCs for cell death by alkaline phosphatase staining.
62. Alkaline phosphatase staining.
  a. Rinse the cells with D-PBS.
  b. Fix the cells with 4% PFA for 20 min at room temperature.
  c. Replace 4% PFA with ddH_2_O.
  d. For a 6-well plate, mix 200 μL alkaline V solution and 200 μL sodium nitrite solution and incubate for 2 min at room temperature.
  e. Add 9 mL ddH_2_O to the above solution.
  f. Add 200 μL naphthol solution to the above solution.
  g. Replace ddH_2_O with 1,000 μL of the above solution.
  h. Incubate the plate overnight in a dark place.
  i. The next day, Remove the staining solution and wash the cells with ddH_2_O.
  j. Remove ddH_2_O immediately and dry the plate.
  k. The area stained in purple indicates the hPSCs that survived during orlistat treatment.
  l. Based on our experiments, optimal dose and duration of orlistat treatment are those which achieve more than 90% efficiency of elimination. Excess dose and duration may be toxic to differentiated cells.
63. After optimal dose and duration of orlistat treatment have been determined by the above steps (on day 5 or later), seed hPSC-CMs in collagen I-coated 150 mm culture dishes.
64. Two days later, prepare StemFit AS103C with orlistat of the determined concentration.
65. Change the culture medium to AS103C supplemented with orlistat and incubate the hPSC-CMs for 24–72 h.
66. Observe the viability of hPSC-CMs by LIVE/DEAD assay.
67. LIVE/DEAD assay.
  a. Prepare staining solution by mixing 7.5 mL D-PBS, 15 μL ethidium homodimer-1 solution, and 3.75 μL calcein-AM solution for a 150 mm culture dish.
  b. Aspirate the culture medium and leave 7.5 mL of the culture medium in the culture dish.
  c. Add the staining solution into the culture dish.
  d. Incubate for 30 min in an incubator at 37°C.
  e. Remove the mixture of culture medium and staining solution and replace with 20 mL AS103C.
  f. Observe live and dead cells under a microscope (Figure 2).

**CRITICAL:** The plate must be protected from light during the procedure for alkaline phosphatase staining.

### Evaluation of hPSCs elimination efficiency by spike cultures

**Timing: 5 days**

This section describes the protocol to determine whether a small molecule of interest can selectively eliminate undifferentiated hPSCs in the presence of other types of cells (somatic cells). This section uses hPSC-CMs purified utilizing the purification method described above. For the use of iMatrix-221, refer to this link for further information.

**Figure 3:**
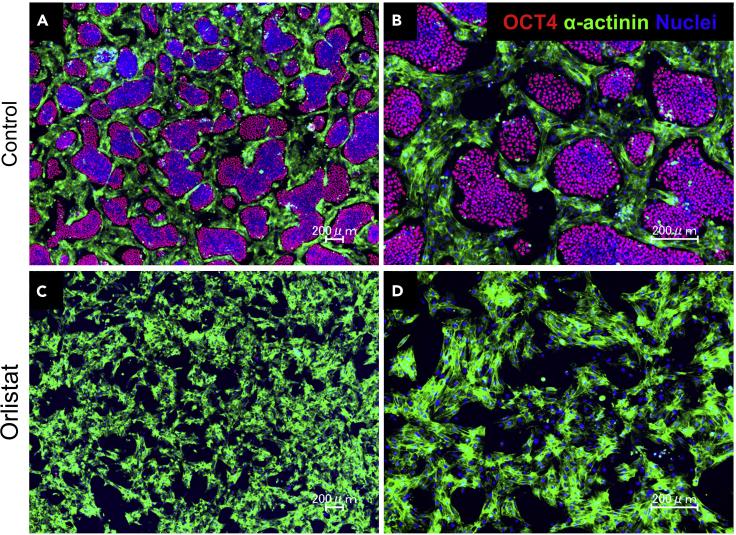
Elimination of undifferentiated hPSCs by spike culture (A and B) Control. (C and D) Orlistat-treated cells.

68. Dilute iMatrix-221 in D-PBS.
  a. For a 12-well plate (culture area: 3.8 cm^2^), dilute 3.8 μL of iMatrix-221 in 1 mL of D-PBS per well.
69. Apply the diluted iMatrix-221 to the wells (for example, 1 mL per well) and incubate the plate for 1 h at 37°C, 3 h at room temperature, or overnight at 4°C.
70. Prepare a suspension of hPSC-CMs as described above with StemFit AS103C + 10 μM Y-27632.
71. Prepare a suspension of undifferentiated hiPSCs as described above with StemFit AS103C plus 10 μM Y-27632.
72. Determine the cell numbers of the hPSC-CMs and undifferentiated hiPSCs.
73. Prepare a suspension containing hPSC-CMs (5 × 10^5^ cells/mL) and undifferentiated hiPSCs (5 × 10^4^ cells/mL).
74. Aspirate diluted iMatrix-221 from the wells and add 1 mL of the cell suspension to each well.
  a. Do not dry the well after aspiration of iMatrix-221.
75. Gently rock the culture plate to ensure that the cells are uniformly dispersed within the well.
76. Gently place the culture plate in an incubator (37°C, 5% CO_2_).
77. On the next day (just before 24 h after seeding the cells), prepare culture medium containing the small molecule of interest.
  a. For example, dilute 0.5 μL of stock solution of orlistat (12 mM) in 1 mL of StemFit AS103C culture medium.
78. 24 h after seeding the cells, aspirate the culture medium and replace it with culture medium containing the small molecules.
79. Incubate at 37°C, 5% CO_2_.
80. Observe the cell status under a phase-contrast microscope every 24 h. If undifferentiated hiPSCs are successfully eliminated, only beating CMs can be observed.
81. 72 h after initiation of the treatment, use the evaluation method of your choice, such as immunocytochemistry or flow cytometry.
82. Immunocytochemistry.
  a. Rinse the cells with D-PBS and fix with ice-cold 4% PFA for 15–20 min at room temperature.
  b. Rinse the cells with D-PBS and treat with 0.1% Triton X-100 diluted in D-PBS for 5–15 min.
  c. Treat the cells with ImmunoBlock for 30 min.
  d. Dilute primary antibodies against OCT4 (Abcam, ab200834, 1:200) and α-actinin (Sigma-Aldrich, A7811, 1:200) with ImmunoBlock reagent.
  e. Apply the diluted antibodies to the cells and incubate overnight at 4°C.
  f. Remove the diluted antibodies and rinse the cells twice with D-PBS.
  g. Dilute the secondary antibodies: 1:200 donkey anti-mouse IgG (H+L) highly cross-adsorbed secondary antibody, Alexa Fluor 488 (Thermo Fisher Scientific, A21202) and 1:200 donkey anti-rabbit IgG (H+L) highly cross-adsorbed secondary, Alexa Fluor 546 (Thermo Fisher Scientific, A10040) with ImmunoBlock reagent.
  h. Apply the diluted antibodies to the cells and incubate for 1 h at room temperature.
  i. Remove the secondary antibodies and rinse the cells with D-PBS.
  j. Dilute Hoechst 33342, 1:2,000 in D-PBS.
  k. Apply diluted Hoechst 33342 to the cells and incubate at room temperature for 1 h.
  l. Remove Hoechst 33342 and apply D-PBS to the cells.
  m. Observe the cells under a microscope. If undifferentiated hiPSCs are successfully eliminated, OCT4-positive undifferentiated hiPSCs will disappear, and only α-actinin-positive hPSC-CMs could be observed (Figure 3).

### Metabolic selection of hiPSC-derived neurons with orlistat treatment

**Timing: 7 days**

This section describes orlistat treatment for the removal of residual undifferentiated hPSCs during neuronal differentiation of hiPSCs. Neuronal differentiation was induced by transfection of mRNA based on a previously published protocol (Goparaju et al., 2017). The mRNA was synthesized from 1 μg of a template DNA encoding NEUROG2 and 3′UTR (Goparaju et al., 2017) with an mMESSAGE mMACHINE™ T7 Transcription Kit (Invitrogen, AM1344). *In vitro* transcription was performed at 37°C for 18 h. m7G Cap Analog (NEB, S1411L), and 5-methyl cytidine-5′-triphosphate (TriLink, N1014) and pseudouridine-5′-triphosphate (TriLink, N1019) were included at the reaction to reduce cytotoxicity when they are transfected into the cells. The purified mRNA (∼30 μg) was stored as aliquots at −80°C until further use.

**Figure 4:**
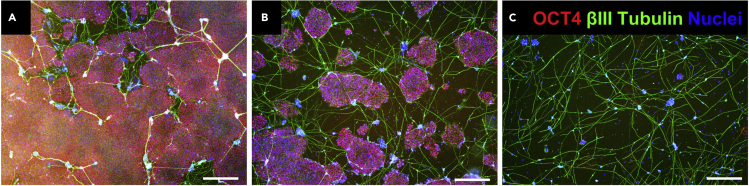
Elimination of residual undifferentiated hPSCs during neuronal differentiation (A) Control. (B) Orlistat treated from day 3 to day 4 post-neuronal differentiation. (C) Orlistat treated from day 3 to day 7 post-neuronal differentiation. (A–C) The cells were fixed on day 7. Scale bars: 500 μm.

This protocol selectively removes residual undifferentiated hPSCs from a population of hiPSC-derived neurons, without the need for cell sorting or reseeding steps (Figure 4). Here, we describe a 24-well plate scale protocol, but this protocol can be adapted to different plate scales by adjusting the number of hiPSCs for seeding and the volume of reagents per well.

83. Dilute iMatrix-511 in D-PBS.
  a. For a 24-well plate (culture area: 2 cm^2^), dilute 1.65 μL of iMatrix-511 in 0.5 mL of D-PBS per well.
84. Apply 0.5 mL of the diluted iMatrix-511 to the wells and incubate the plate for 1 h at 37°C.
85. Prepare StemFit AK02N medium supplemented with 10 μM Y-27632.
86. Prepare a suspension of 2 × 10^5^ cells/mL in StemFit AK02N medium with Y-27632.
87. Aspirate the diluted iMatrix-511 from the wells and apply 500 μL of the cell suspension prepared in the previous step to each well.
  a. Do not dry the well after aspiration of iMatrix-511.
88. The following day (day 0), transfect NEUROG2 synthetic mRNA to induce neural differentiation as follows:
  a. Add 2 μL of Lipofectamine Messenger Max to 50 μL of OPTI-MEM medium and vortex briefly at medium speed. Leave at room temperature (25°C) for 10 min (Solution A).
  b. Add 1 μg of NEUROG2 synthetic mRNA in 50 μL of OPTI-MEM medium and vortex briefly at medium speed. (Solution B).
  c. Mix solutions A and B and vortex briefly at medium speed. Incubate the mixture at room temperature for 4 min.
  d. Slowly drop the mixture onto the cells.
  e. At 3 h post-transfection, change the medium to 500 μL of StemFit AK02N with 10 μM Y-27632.
89. After 1 h, repeat steps a–e.
90. On day 1, transfect the synthetic mRNA once following steps a–e.
  a. Most of the hiPSCs (∼90%) should be transfected with the synthetic mRNA and can be differentiated into mature neurons (Goparaju et al., 2017). The non-transfected cells occupy a small population, but they will continue to proliferate in an undifferentiated state.
91. On day 2, change the medium to 500 μL of StemFit AK02N.
92. On day 3, change the medium to 500 μL of StemFit AK02N with 6 μM orlistat. Treat the cells with orlistat for 4 days. Change half of the medium daily from day 3 to day 7. Orlistat causes cell death of only undifferentiated hPSCs.
93. On day 7, most of the cells should be differentiated into neurons. Perform your desired experiments (e.g., immunofluorescence, RT-PCR, electrophysiology, etc.).
94. Immunocytochemistry.
  a. Rinse the cells with D-PBS and fix with ice-cold 4% PFA for 15–20 min at room temperature.
  b. Rinse the cells with D-PBS and treat with 0.1% Triton X-100 diluted in D-PBS for 5–15 min.
  c. Treat the cells with ImmunoBlock for 30 min.
  d. Dilute the primary antibodies against OCT4 (Santa Cruz Biotechnology, sc-5279, 1:1000) and βIII-tubulin (Cell Signaling, 5666, 1:1000) with ImmunoBlock.
  e. Apply the diluted antibodies to the cells and incubate for 3 h at room temperature.
  f. Remove the diluted antibodies and rinse the cells twice with D-PBS.
  g. Dilute the secondary antibodies (donkey anti-mouse IgG secondary antibody, Alexa Fluor 594 (Thermo Fisher Scientific, A21203, 1/500) and goat anti-rabbit IgG, Alexa Fluor 488 (A11008, 1/500; Thermo Fisher Scientific).
  h. Apply the diluted antibodies to the cells and incubate for 1 h at room temperature.
  i. Remove the secondary antibodies and rinse the cells with D-PBS.
  j. Stain the nuclei with DAPI (Dojindo, D523) for 5 min at room temperature.
  k. Remove DAPI and apply D-PBS to the cells.
  l. Observe the cells under a microscope.

**CRITICAL:** Treatment with orlistat for 1 d can cause cell death, but such a short treatment is not sufficient to remove all undifferentiated hPSCs.

**CRITICAL:** Early treatment with orlistat (e.g., from day 1 post neuronal induction) can cause cell death of not only undifferentiated hPSCs but also differentiating cells. Treatment from day 2 or 3 is effective for selectively removing undifferentiated hPSCs.

## Expected outcomes

When undifferentiated hPSCs are successfully removed, the OCT4-positive cells will disappear (Figures 1 and 2). This protocol has been validated using multiple hPSC lines. The use of hPSC-CMs purified by this protocol prevented the common problem of tumor formation in a mouse teratoma model, and therefore would be suitable for use in heart regenerative medicine. Although we have not applied this method to other differentiation protocols that use mRNAs, we believe that this method could be applied to these protocols.

## Limitations

Some hPSC lines have a limited ability to differentiate into CMs. In addition, although we have confirmed that hPSC-CMs express FASN at significantly lower levels than undifferentiated hiPSCs, it is unclear whether inhibition of FASN affects the function of hPSC-CMs.

## Troubleshooting

### Problem 1

hPSCs do not differentiate efficiently. Differentiation to hPSC-CMs.

### Potential solution

Some hPSC lines have a limited ability to differentiate into CMs. Validate differentiation efficiency by testing various numbers of cells when plating and using several concentrations of CHIR-99021.

### Problem 2

hPSC-CMs detach from collagen-coated dishes during purification. Metabolic selection of hPSC-CMs.

### Potential solution

Coat the collagen-coated culture dishes with fibronectin. Dilute fibronectin in PBS (SIGMA, F2006, 1:100) and apply diluted fibronectin to collagen-coated 150-mm culture dishes (20 mL per dish). Incubate the dishes overnight at 37°C.

### Problem 3

Poor colony formation in the control group while evaluating the elimination efficiency of hPSCs by spike culture. Evaluation of hPSCs elimination efficiency by spike cultures.

### Potential solution

Increase the ratio of hPSCs to 20%–30%.

### Problem 4

Orlistat treatment does not effectively eliminate undifferentiated hPSCs. Evaluation of the optimal dose and duration of orlistat treatment.

### Potential solution

Check the composition of the culture medium. Lipids in the culture medium may lower the dependency to *de novo* fatty acid synthesis in hPSCs and thus perturbs selection efficiency.

### Problem 5

Differentiated cells of interest are lost by orlistat treatment. Metabolic selection of hPSC-CMs, evaluation of hPSCs elimination efficiency by spike cultures, metabolic selection of hiPSC-derived neurons with orlistat treatment.

### Potential solution

Determine the optimal concentration and treatment period of orlistat again with the differentiated cells of interest.

## Resource availability

### Lead contact

Further information and requests for resources and reagents should be directed to and will be fulfilled by the lead contact, Shugo Tohyama (shugotohyama@keio.jp).

### Materials availability

This study did not generate any new unique reagents.

## Data Availability

This study did not generate/analyze any datasets or code.
